# Real-time PCR detection of Human Herpesvirus 1-5 in patients lacking clinical signs of a viral CNS infection

**DOI:** 10.1186/1471-2334-11-220

**Published:** 2011-08-17

**Authors:** Birgitta Sundén, Marie Larsson, Tina Falkeborn, Jakob Paues, Urban Forsum, Magnus Lindh, Liselotte Ydrenius, Britt Åkerlind, Lena Serrander

**Affiliations:** 1 Div Clinical Microbiology, Department of Clinical and Experimental Medicine, Linköping University, SE-581 85 Linköping, Sweden; 2 Div Infectious Diseases, Department of Clinical and Experimental Medicine, Linköping University, SE-581 85 Linköping, Sweden; 3 Department of Infectious Diseases, Section for Clinical Virology, University of Gothenburg, SE- 413 46 Gothenburg, Sweden

## Abstract

**Background:**

Infections of the central nervous system (CNS) with herpes- or enterovirus can be self-limiting and benign, but occasionally result in severe and fatal disease. The polymerase chain reaction (PCR) has revolutionized the diagnostics of viral pathogens, and by multiple displacement amplification (MDA) prior to real-time PCR the sensitivity might be further enhanced. The aim of this study was to investigate if herpes- or enterovirus can be detected in cerebrospinal fluid (CSF) from patients without symptoms.

**Methods:**

Cerebrospinal fluid (CSF) samples from 373 patients lacking typical symptoms of viral CNS infection were analysed by real-time PCR targeting herpesviruses or enteroviruses with or without prior MDA.

**Results:**

In total, virus was detected in 17 patients (4%). Epstein-Barr virus (EBV) was most commonly detected, in general from patients with other conditions (e.g. infections, cerebral hemorrhage). MDA satisfactorily amplified viral DNA in the absence of human nucleic acids, but showed poor amplification capacity for viral DNA in CSF samples, and did not increase the sensitivity for herpes virus-detection with our methodology.

**Conclusions:**

Viral pathogens are rarely detected in CSF from patients without signs of CNS infection, supporting the view that real-time PCR is a highly specific method to detect symptomatic CNS-infection caused by these viruses. However, EBV may be subclinically reactivated due to other pathological conditions in the CNS.

## Background

Herpes viruses are neurotropic and may cause encephalitis, a serious condition with mortality rates around 10-20% overall mortality and up to 20-30% for untreated Herpes simplex-1 (HSV-1) encephalitis. Therefore the detection of these viruses in patients with suspected viral infections of the central nervous system (CNS) is well studied [1-7]. Due to their neurotropism, alpha-herpesviridae may reside latently in neurons after the primary infection. Dormant viruses may subsequently reactivate and cause symptoms of meningoencephalitis. However, it is not known if viral particles are shed into the cerebrospinal fluid (CSF) during the latent phase, since few studies have investigated the presence of herpes viruses in the CSF of patients lacking symptoms of viral CNS infection [8,9]. In an earlier study, Davies et al. showed that viral nucleic acid (NA) could be detected by PCR in 5% of patients classified as having a low probability of CNS infection [8].

Although PCR is a highly sensitive method for detection it may not always be sensitive enough for identification of viral DNA in CSF, due to the fact that viral shedding from latent infection may be very low. Multiple displacement amplification (MDA), a whole genome amplification (WGA) method for isothermal uniform amplification of total DNA, might serve to increase the sensitivity of the polymerase chain reaction (PCR)[10,11]. To our knowledge only a few studies have used MDA in connection with viral detection [12-14].

The aims of this project were to investigate the possibility to detect viral nucleic acid in CSF from patients without clinical symptoms or signs of viral infection and if herpes- or enterovirus might cause symptoms mistaken for bacterial CNS infections. Attempts were made to increase the sensitivity by amplifying total DNA with MDA prior to real-time PCR.

## Methods

### Patients

Samples of CSF from 373 patients from Östergötland County were included in the study (Table 1). No viral analysis had been performed on the majority of these samples. Samples from two groups of patients were studied. Patient group 1 included samples from 167 patients that were sent to the clinical microbiological laboratory at the Linköping University Hospital, for bacterial culture because of a suspected bacterial infection in the CNS. The patients in this group had symptoms ranging from low-grade fever with or without increased white blood cells (WBC) in blood or CSF, to acute onset of decreased consciousness, confusion and sepsis. These samples were collected consecutively between January and September 2008. Patient group 2 consisted of 206 patients, who's CSF, collected consecutively from January to March and June to July 2007, had been previously analyzed serologically for neuroborreliosis. Many of these patients had diffuse symptoms such as persistent or intermittent headaches; others had a history or symptoms that can be indicative of neuroborreliosis. Four patients out of the 206 (2%) in this group were diagnosed with neuroborreliosis using the IDEIA™ Lyme Neuroborreliosis EIA kit (Oxoid Ltd, Ely UK).

**Table 1:** Sample population of patients

	Age		Gender distribution		Total
	Median	Range	Men (%)	Women (%)	Patients
Patient group 1	55	0-86	79 (47)	88 (53)	167
Patient group 2	49	3-84	92 (45)	114 (55)	206

Patient group 1 consisted of samples that were sent for bacterial culture because of a suspected bacterial infection in the CNS. Patient group 2 consisted of samples that had been previously analyzed for neuroborreliosis.

### Viral nucleic acid extraction

Total nucleic acid was extracted from 200 μl CSF using MagAttract Virus Mini M48 kit (Qiagen, Solna, Sweden) according to manufacturer's instructions and eluted in 75 μl. All samples were frozen after extraction and after MDA-treatment, when performed.

### MDA

GenomiPhi V2 DNA Amplification kit (GenomiPhi, GE Healthcare, Stockholm, Sweden) was used according to manufacturer's instructions on 76 samples, with modification for using 10 μl of DNA extract without sample buffer to maximize initial DNA content. The amplification capacity of MDA for each virus was evaluated by mixing positive herpesvirus DNA controls (Vircell, Santa Fé, Spain) in nuclease-free water or CSF at various concentrations. Real-time PCR results were compared for samples with and without MDA treatment. For this evaluation GenomiPhi was used with either 5 μl template mixed in 5 μl sample buffer, or with 10 μl template without sample buffer. REPLI-g Mini kit (Qiagen) was also used for this evaluation according to the manufacturers' recommendations with 5 μl template.

### Real-time PCR

Real-time PCR reactions were performed on Rotor-Gene (Corbette Life Science, Sydney, Australia). Extracted samples with and without MDA treatment were thawed and analyzed in parallel. HSV-1 and HSV-2 were analyzed with 1X QuantiTect SYBR Green Master Mix (Qiagen), 5 μl template in a final volume of 25 μl. PCR for detection of Varicella zoster virus (VZV) followed the same protocol as HSV-1 and HSV-2, but using 1X QuantiFast Probe Master Mix (Qiagen). Sample results were analyzed for cycle threshold values and melting curve analysis for HSV-1 and HSV-2. Commercial kits Artus EBV RG PCR kit V1 (Qiagen) and Artus CMV RG PCR kit V1 (Qiagen) were used according to the manufacturer's instructions. Enterovirus was detected with RNA UltraSense™ One-Step Quantitative RT-PCR System (Invitrogen, Paisly, United Kingdom) according to manufacturer's instructions, with 1X RNA UltraSense™ Reaction mix, 3 mM MgSO_4_, 1.25 μl UltraSense™Enzyme mix and 10 μl template to a final volume of 25 μl. Primers and probes for detection are shown in Table 2. In Patient group 1, real-time PCR analysis included 167 samples for Human herpes virus (HHV) 1 to 5 and samples from 93 patients for enterovirus. In Patient group 2, corresponding PCR analysis included 206 samples for HHV 1 to 4, 171 samples for cytomegalovirus (CMV) and samples from 110 patients for enterovirus.

**Table 2:** Primers and probes for HSV-1, HSV-2, VZV and enterovirus

Virus	Primer or probe	Sequence (5'-3')	Primer/probe concentration
HSV-1^1^	Forward	CCATACCGACCACACCGACGA	400 nM
	Reverse	CATACCGGAACGCACCACAC	400 nM
HSV-2^1^	Forward	TCAGCCCATCCTCCTTCGGC	400 nM
	Reverse	GCGCGGTCCCAGATCGG	400 nM
VZV	Forward	TGCAGGGCATGGCTCAGT	400 nM
	Reverse	CCCAAGAACCACATGTCCAAC	400 nM
	Probe	TCCAGGGACTTGGGACC	200 nM
Enterovirus	Forward	CCTGAATGCGGCTAATCCYAAC^2^	400 nM
	Reverse	CRATTGTCACCATAAGCAGCCA^3^	900 nM
	Probe	CCCAAAGTAGTCGGTTCCGCCACAGA	150 nM

^1 ^Primers for HSV-1 and modified primers for HSV-2 from Franzén-Röhl 2008^7^.

^2 ^Degenerate primer, Y = T or C

^3 ^Degenerate primer, R = G or A.

## Results

### Real-time PCR

Out of totally 167 patients in Patient group 1, with suspected bacterial CNS infection, viral NA was detected in the CSF of 15 patients using real-time PCR (Table 3). Epstin-Barr virus (EBV) was the most common viral DNA detected (9 patients). As described in Table 4, CNS symptoms was due to non-infectious causes in several cases; subarachnoid or cerebellar hemorrhage (n = 5), tumors (n = 3) or was explained by bacterial infection (n = 4) or shunt dysfunction (n = 2). In two cases, EBV DNA was detected together with HSV-1 or VZV DNA. Among the 206 patients with possible neuroborreliosis, (Patient group 2), viral NA (EBV) was detected in two cases.

**Table 3:** Real-time PCR detected viral DNA in the two patient groups

Virus	Patient group 1 (167 tot)		Patient group 2 (206 tot)	
	Positive	Percent	Positive	Percent
HSV-1	2	1%	0	0%
HSV-2	1	0.6%	0	0%
VZV	1	0.6%	0	0%
EBV	7	4%	2	1%
CMV	1	0.6%	0	0%
EV	1	0.6%	0	0%
HSV-2 + EBV	1	0.6%	0	0%
VZV + EBV	1	0.6%	0	0%

Total positive patients	15	9%	2	1%

**Table 4:** Clinical and laboratory finding in patients positive for virus in CSF

						Symptoms		blood		CSF				
**Pat**	**Age**	**Sex**	**Results**		**Admission cause**	**Enceph**	**Mening**	**WBC**	**CRP**	**WBC**	**Poly**	**Mono**	**Diagnosis**	**AVT**

1	77	M		EBV	acute onset, reduced conciousness	No	No	11	62	5	2	3	SAH, bacterial infection	

2	64	F	VZV	EBV	fever, decreased consciousness	No	No	9	45	427	170	257	Brain tumor	

3	53	M	HSV2	EBV	fever, decreased consciousness	No	Yes	11	109	135	27	108	Brain hemorrhage, meningitis	

4	53	M	HSV1		headache, personality change	Yes	No	8	< 10	0.5	0.4	0.1	Hydrocephalus/encephalitis	

5	82	M		CMV	fever, post op cerebi	No	No	17	63	21	5	16	Meningioma, infection	

6	86	F		EV	fever, decreased consciousness	Yes	Yes	10	140	1.4	0	1.4	Herpes zoster, bacteremia	yes

7	61	F		EBV	post op double vision, CSF leakage	No	No	10	< 10	1315	515	800	Meningioma, post-op meningitis	

8	70	F	HSV2		sudden headache	No	Yes	6	11	103	18.6	84	HSV2 meningitis	

9	58	M	HSV1		acute confusion, dysphasia	Yes	No	16	< 10	27	0.6	26	HSV1 encephalitis	yes

10	52	F		EBV	dizziness, vomiting, headache	No	No	18	89	104	n.d	n.d.	SAH	

11	56	M	VZV		Rash, decreased consciousness	No	Yes	6	111	26	0	26	varicella meningitis	yes

12	65	M		EBV	Fever, post op cerebri	No	No	11	32	150	2	148	Spinal metastasis, *S. aureus *inf	

13	65	M		EBV	ventricular shunt dysfunction	No	No	5.5	< 10	109	3	106	ventricular shunt dysfunction	

14	63	M		EBV	fever post-op	No	No	9	< 10	1.7	0.1	1.6	SAH	

15	54	F		EBV	acute headache	No	No	16	98	96	n.d.	n.d	SAH, pneumonia	

16	60	F		EBV	dizziness, arm weakness	No	No	6	< 10	1	0.2	0.6	cerebrovascular disease	

17	40	F		EBV	double vision, dysphasia, headache	Yes	No	14	< 10	514	10	504	Borrelia	yes

SAH: subarachnoidal hemorrhage, Enceph: encephalitis, Mening: meningitis, WBC: white blood cells, CSF: cerebrospinal fluid, CRP: C-reactive protein, Poly: polymorphonuclear cells, Mono: mononuclear cells, AVT: antiviral treatment, n.d.: not determined.

### MDA

Five samples (one each of HSV-1, HSV-2 and CMV, and two EBV) in which viral DNA was detected by PCR (without MDA) were negative by PCR after MDA. Conversely, one sample from Patient group 1 was positive for HHV DNA only after MDA amplification. This sample showed a very weakly positive signal after MDA treatment, suggesting that the amount of viral NA was close to the assay cut off. EBV was detected in two samples both with and without MDA. These two EBV samples displayed higher Ct values for MDA products, suggesting that MDA did not increase the sensitivity for viral DNA. Since MDA did not increase the sensitivity for detecting HHV it was not performed on more than 76 samples.

A reason why MDA-treatment did not increase sensitivity could be that viral DNA is scarce in CSF compared to the amount of human genomic DNA. Therefore we studied the amplification of viral DNA from infected cell cultures. MDA-treated viral DNA diluted in CSF compared to water showed a poorer amplification for viral DNA, particularly at lower concentrations. These results suggest that MDA negatively discriminates viral DNA in presence of human nucleic acid.

## Discussion

In this study we found no support for low-grade or intermittent shedding of latent HHV DNA into the CSF in patients without signs of viral CNS infection. The identification of viral NA in samples sent for bacterial culture is interesting and probably reflects that clinical symptoms are not easily categorised as due to bacterial or viral infection in the CNS. Probably, bacterial culture is requested also in the absence of typical meningitis in patients who are old, have a complex medical history or are immunologically suppressed.

Viral NA was detected in 15 samples (8%) sent for bacterial culture on the suspicion of possible bacterial CNS infection (Patient group 1). HSV-1, HSV-2 or VZV DNA was detected in 6 of these cases. Five of these had a clinical presentation compatible with viral meningitis or encephalitis, while such symptoms were absent in one case (Pat no 2). CMV was detected in one patient, but the clinical picture did not support CMV-caused infection. EBV DNA was detected in 9 patients in Group 1, but only few of them had signs of CNS infection and none had symptoms typical for EBV infection (fever, mononucleosis). It has been described that EBV can cause CNS infections by itself, particularly in immunocompromised patients [15-18] but EBV DNA has also been detected in CSF and synnovial fluid from patients without likely EBV-infection [8,9,19]. Our results support the latter, but it is important to note that our material primarily is from patients without suspected viral infection.

It is to our knowledge not yet known if EBV DNA detected in the CSF origins from the CNS itself or from infiltrative white blood cells due to inflammation. The explanation that EBV is borne by WBCs may be favoured, since we could only detect EBV in patients with other traumatic and/or inflammatory processes in the CNS, see Table 4.

Viral NA (EBV) was detected in only two of the samples in Patient group 2 (0.5%). One patient had a clinical presentation characteristic for neuroborreliosis, and did not have fever or other signs of EBV infection. The other patient had no signs of infection but suffered from cerebrovascular disease. The more rare detection of viral NA in Patient group 2 was not surprising because the majority of these samples were collected from patients with diffuse symptoms of long duration, drawn because the clinician wanted to exclude the possibility of neuroborreliosis.

Enterovirus infections in the CNS are common and may present as meningitis in a manner that may mimic bacterial infection. Still, enterovirus RNA was detected in only one positive sample in Patient group 1 and the sample was very weakly reactive. The patient did not have typical symptoms for enterovirus infection, nor increased white blood cells in the CSF.

The absence of detectable viral NA suggests that viruses were not present in the CSF or that our methods for detection are still not sensitive enough. Most real-time PCR methods detect 1 to 10 viral particles/PCR reaction, but the concentration of these viruses in CSF during subclinical infection might be very low. A higher sensitivity could probably be achieved by using larger CSF extraction volumes (5-20 ml), but in general this is not practically possible.

In our study, MDA amplified viral DNA satisfactorily in the absence of other nucleic acids, but showed poor amplification capacity for viral detection in CSF and did not overall increase the sensitivity for HHV detection with our methodology. A possible explanation why viral DNA was not amplified could be the viruses' shorter genome, the nature of viral DNA, or as reported in a study of *H. pylori *DNA, impact from excess of human genomic DNA [20]. The reportedly good performance of MDA amplification for human papilloma virus (HPV) and human immunodeficiency virus (HIV) may be explained by the fact that in these cases, viral DNA can be integrated into the human genome [13,21]. This suggests that HHV DNA, which is not inserted into the human genome, is discriminated by this method.

## Conclusions

In summary, our results show that viral infection, in particular HSV and VZV, should be suspected in patients with putative bacterial CNS infection. Conversly, patients presenting clinical symptoms suggesting HSV-1 encephalitis may have bacterial cause *e.g*. listerial infections [22,23]. The findings also indicate that MDA does not serve to enhance the performance of real-time PCR, which alone is a both highly sensitive and specific method for detecting viral CNS infections.

## Abbreviations

CNS: central nervous system; CSF: cerebrospinal fluid; HSV: herpes simplex virus; HHV: human herpesvirus; EBV: Epstein-Barr virus; CMV: cytomegalovirus; WGA: whole genome amplification; MDA: multiple displacement amplification; NA: nucleic acids; WBC: white blood cells.

## Competing interests

The authors declare that they have no competing interests.

## Authors' contributions

BS carried out the molecular analysis and design, performed the initial data analysis and drafted the manuscript. TF participated in the discussions and the molecular work. JP participated in the analysis of patient data and helped to draft the manuscript. UF provided patient samples and helped with the design of the study and helped to draft the paper. ML participated in the analysis of data and helped to draft the paper. LY participated in the analysis of data and the critical reading of the paper. BÅ provided patient samples, participated in the design of the study and the draft of the paper. LS conceived the study, and participated in its design and coordination and helped to draft the manuscript. All authors read and approved the final manuscript.

## Pre-publication history

The pre-publication history for this paper can be accessed here:

http://www.biomedcentral.com/1471-2334/11/220/prepub
